# Exploring Chiropractic Healthcare in Hong Kong: Sick Leave Certification Dilemma

**DOI:** 10.7759/cureus.52957

**Published:** 2024-01-25

**Authors:** Wai Ting Lee, Eric Chun-Pu Chu, Kary Lam, Rick Lau, Jacky Yeung, Kristy Yau, Cherie Chau

**Affiliations:** 1 Integrative/Complementary Medicine, Chiropractic and Physiotherapy Centre, New York Medical Group, Hong Kong, CHN; 2 Integrative/Complementary Medicine, Chiropractic Doctors Association of Hong Kong, Hong Kong, HKG; 3 Therapeutics, Chiropractic Doctors Association of Hong Kong, Hong Kong, HKG; 4 Integrative/Complementary Medicine, Chiropractic and Physiotherapy Centre, New York Medical Group, Hong Kong, HKG; 5 Integrative/Complementary Medicine, EC Healthcare, Hong Kong, HKG

**Keywords:** sick leaves, chiropractic, public heath, chiropractic therapy, hong kong chiropractor

## Abstract

Objective

This study aims to investigate the characteristics of chiropractic patients in Hong Kong, their experiences with chiropractic care, and their perspectives on chiropractors' authority over sick leave certificates.

Method

A cross-sectional survey was conducted among individuals receiving chiropractic treatment in Hong Kong. Data were collected through an online survey from May 11 to August 8, 2023, and descriptive analysis was employed to examine patient demographics, treatment effectiveness, and views on chiropractic sick leave authorization. A total of 522 valid responses were received.

Result

Among respondents, back pain was the primary reason for seeking chiropractic care, with many experiencing rapid relief and high satisfaction. However, many patients initially consulted other healthcare professionals, indicating potential integration challenges. Lengthy orthopedic wait times in Hong Kong highlight the need for chiropractic care. Concerns arose over chiropractors' inability to issue sick leave certificates, impacting patient convenience, treatment effectiveness, finances, and emotional well-being. Allowing chiropractors to authorize sick leave, with proper regulation, could address these issues.

Conclusion

In conclusion, this study underscores chiropractic care's potential in Hong Kong's healthcare system and suggests that recognizing chiropractors' role in sick leave authorization can enhance comprehensive patient care.

## Introduction

Chiropractors in Hong Kong have gained widespread popularity as primary healthcare providers by offering non-invasive and drug-free treatments for managing neuromusculoskeletal complaints [1,2]. Since 1993, chiropractors in Hong Kong have gained legal recognition as primary healthcare providers specializing in the diagnosis and management of neuromusculoskeletal complaints [3]. As the profession continues to grow, they have become essential members of the city's primary healthcare system, delivering effective chiropractic interventions and providing holistic education and counseling to the general public [4].

Despite their efficacy and proficiency in managing neuromusculoskeletal disorders, chiropractors are currently excluded from the authorization to issue sick leave certificates, limiting their ability to grant patients leave from work or school for recovery. According to Hong Kong's labor law, only medical practitioners, Chinese medicine practitioners, and dentists are authorized to issue sick leave for employees [5]. The authorization of sick leave certificates for chiropractors has not been extensively discussed within the Hong Kong government. The only known initiative from the government was an interdepartmental working group established in 2005; however, no conclusive outcomes were reached [6].

Given this limitation, individuals undergoing chiropractic care will find it necessary to consult other healthcare professionals in order to obtain sick certificates. This additional process is anticipated to potentially impose burdens on the patients, either in terms of their financial commitments or physical well-being [7].

Prior reviews have been conducted to analyze the demographic attributes of individuals who undergo chiropractic care [8,9]. Nevertheless, there is limited information available concerning treatment effectiveness, opinions, and comments regarding the authority of chiropractors in relation to sick leave. This study aims to delve into the present state of chiropractic patient characteristics while gathering their perspectives on chiropractors holding the authority to make decisions about sick leave.

## Materials and methods

Design

The study utilizes a cross-sectional survey design to investigate the current characteristics of chiropractic patients and their perspectives regarding chiropractors’ authority over sick leave certificates. The study received approval from the ethics committee of the Chiropractic Doctors' Association of Hong Kong (Causeway Bay, Hong Kong; IRB ID: CDA20230102), and written consent was waived since consent was implied by the return of the completed survey. To ensure respondent anonymity, names and other identifying information were not collected.

Setting

The target population of this study comprises individuals currently receiving chiropractic treatment in Hong Kong.

Instrument

A comprehensive survey questionnaire was formulated bilingually in Chinese and English to investigate various aspects, including the characteristics of chiropractic patients, the effectiveness of treatments, and viewpoints on chiropractic sick leave authorization. This questionnaire was informed by references from earlier reviews [8,9]. It comprised 20 questions spread across three sections: demographic information, experiences related to chiropractic care, and perspectives concerning chiropractic sick leave authorization (see Appendices 1).

Data sources

The data for this study were collected by distributing a survey using the Google Forms platform. The survey was distributed to all registered chiropractors in Hong Kong through their respective chiropractic associations then the survey was circulated to all individuals who visited chiropractic clinics by either chiropractors or clinic staff in Hong Kong between May 11, 2023 and August 8, 2023. The completed survey forms served as the sole source of data for this study. Data were extracted from the survey responses and input into a spreadsheet format for further analysis.

Bias

To ensure the clarity of survey items and estimate the time required to complete the questionnaire, a pilot test was conducted with 10 chiropractic patients. All participants completed the survey within 10 minutes, and the pilot test indicated that the questions were clear and easily understandable. No adjustments were deemed necessary.

Sample size

The sample size was determined using a rule-of-thumb estimation, suggesting that each survey item should have a minimum of five respondents. As a result, a total of 100 respondents were required for this study.

Analysis

The data extracted from the questionnaires were analyzed using Microsoft Excel. Frequency tables and descriptive statistics were employed to investigate the present characteristics of chiropractic patients, the effectiveness of treatment, and perspectives on chiropractic sick leave authority.

## Results

The survey was administered online through a Google Forms link from May 11 to August 8, 2023. Out of the 524 responses received, two were incomplete, resulting in 522 valid responses (99.6% completion rate).

Respondents demographics

The participants were distributed among different age groups. The largest proportion of participants fell within the 40-49 age bracket (23.8%, n=124), followed by those aged 30-39 (20.9%, n=109). Respondents aged 20-29, 50-59, 60+, under 15, and 15-19 accounted for 20.2% (n=105), 14.8% (n=77), 14.4% (n=75), 3.6% (n=19), and 2.3% (n=12), respectively. Within the participant pool, it was found that 59.4% (n=309) of the respondents identified as females, while 40.4% (n=210) were identified as males.

Among the respondents, a majority (78.7%, n=411) were engaged in economic activities. Retired individuals constituted 7.7% (n=40) of the participants, while students and homemakers represented 7.1% (n=37) and 4.4% (n=23), respectively. In terms of educational background, the survey results indicate that a majority of respondents (63.3%, n=329) have attained post-secondary education. This is followed by secondary education (33.3%, n=173) and primary education and below (3.5%, n=18). Table 1 presents the demographic information of the respondents.

**Table 1:** Demographical information by the respondents n= number of respondents

Characteristic		n=522 (%)
Age		
	Under 15	19 (3.6)
	15-19	12 (2.3)
	20-29	105 (20.2)
	30-39	109 (20.9)
	40-49	124 (23.8)
	50-59	77 (14.8)
	60+	75 (14.4)
Gender		
	Male	210 (40.4)
	Female	309 (59.4)
Occupation		
	Student	37 (7.1)
	Full Time Employed	339 (64.9)
	Part Time Employed	32 (6.1)
	Self Employed	40 (7.7)
	Unemployed	6 (1.1)
	Home-makers	23 (4.4)
	Retired	40 (7.7)
	Other	5 (1)
Level of Education		
	Primary and Below	18 (3.5)
	Secondary	173 (33.3)
	Post-Secondary	329 (63.3)

Chiropractic treatment

Among the respondents who had received chiropractic treatment over the past 12 months, 28.8% (n=148) reported receiving treatment 11 times or more, 27.6% (n=142) received treatment one to two times, 15.6% (n=80) received treatment five to six times, 14.2% (n=73) received treatment seven to 10 times, and 13.8% (n=71) received treatment three to four times. Table 2 illustrates the number of chiropractic treatments received during the past 12 months by the respondents.

**Table 2:** Number of chiropractic treatments received during the past 12 months n= number of respondents

Number of visits		n=522 (%)
1-2 times		142 (27.6)
3-4 times		71 (13.8)
5-6 times		80 (15.6)
7-10 times		73 (14.2)
11+ times		148 (28.8)

The most frequently mentioned reasons for selecting chiropractic treatment included “referral from relatives/friends” (52.9%, n=276), “wanted to see about whether chiropractic treatment could alleviate their condition” (51.5%, n=269), “information from various media sources” (34.3%, (n=179), “recommendation from other healthcare professionals” (24.3%, n=127), “insurance coverage” (8.8%, n=46), and “other factors” (6.5%, n=36). Table 3 shows the reasons for choosing chiropractic treatment by the respondents.

**Table 3:** Reason for choosing chiropractic treatments by respondent n= number of respondents

Reason for choosing chiropractic treatment		n=522 (%)
Relatives/friends’ referral		276 (52.9)
Referred by other healthcare professionals		127 (24.3)
Wanted to see whether chiropractic treatment could help alleviate their illness		269 (51.5)
Got to know chiropractors from different media		179 (34.3)
Chiropractic treatment was covered by the insurance plan		46 (8.8)
Others		36 (6.5)

The primary reasons cited by the respondents for seeking chiropractic treatment were “back pain” (37.2%, n=194), “neck pain” (29.1%, n=152), “limb pain” (12.5%, n=65), “other issues” (10%, n=52), “shoulder pain” (5.9%, n=31), and “headaches” (5.4%, n=28). Furthermore, the body parts most frequently mentioned as having received treatment were the “back” (60.6%, n=314), “neck” (56.9%, n=295), “waist” (53.3%, n=267), “limbs” (45.1%, n=234), “shoulders” (30.5%, n=158), and “head” (14.7%, n=76). Table 4 describes the reason for seeking chiropractic treatments and the part of the body that has received treatment by the respondents.

**Table 4:** Reason for seeking chiropractic treatments and part of the body having received treatment by the respondent n= number of respondents

Reason for seeking chiropractic treatment		n=522 (%)
Back pain		194 (37.2)
Neck pain		152 (29.1)
Headache		28 (5.4)
Upper limb pain		15 (2.9)
Lower limb pain		50 (9.6)
Shoulder pain		31 (5.9)
Others		52 (10)
Part of the body having received chiropractic treatment		n=522(%)
Waist		267 (53.3)
Back		314 (60.6)
Neck		295 (56.9)
Lower limbs		138 (26.6)
Upper limbs		96 (18.5)
Shoulder		158 (30.5)
Head		76 (14.7)

The time intervals between injury or illness and respondents' first chiropractic treatment varied significantly. Notably, 24.8% (n=127) sought treatment within one to two weeks, and 22.5% (n=115) did so in less than one week. Additionally, 15.6% (n=80) waited for two to four weeks, while 10.7% (n=55) delayed treatment for four weeks to less than three months. In contrast, 16.8% (n=86) waited six months or more before their first chiropractic treatment. Table 5 shows the time lag between injury/illness and the first chiropractic treatment by respondents.

**Table 5:** Time lag between injury/illness and first chiropractic treatment n= number of respondents

Time lag		n=522 (%)
Less than 1 week		115 (22.5)
1 week to less than 2 weeks		127 (24.8)
2 weeks to less than 4 weeks		80 (15.6)
4 weeks to less than 3 months		55 (10.7)
3 months to less than 6 months		49 (9.6)
6 months above		86 (16.8)

Treatment effectiveness

Respondents' assessments of their conditions' impact on daily activities and work-related tasks varied. A significant portion (32.3%, n=168) reported a slight impact, while 28.8% (n=150) experienced a moderate influence. Moreover, 16.2% (n=84) indicated a significant impact, signifying substantial hindrance and 11.3% (n=59) reported being unable to perform daily activities or work-related tasks due to their conditions.

The majority of the respondents (72.1%, n=373) sought treatment from other healthcare professionals before turning to chiropractic care. Specifically, 55.1% (n=255) of the respondents consulted a physiotherapist, 53.3% (n=247) visited a general medical practitioner, 52.7% (n=244) sought help from a traditional Chinese medicine practitioner, 47.7% (n=221) opted for acupuncture, 41.5% (n=192) consulted with a specialist, and 27% (n=125) sought assistance from a bone setter. Table 6 presents whether respondents had received other kinds of treatment before receiving chiropractic treatment.

**Table 6:** Whether respondents had received other kinds of treatment before receiving chiropractic treatment n= number of respondents

Whether respondents received treatment before chiropractic treatment		n=522 (%)
Yes		373 (72.1)
No		144 (27.9)
Types of treatment received		n=522(%)
General medical practitioner of Western medicine		247 (53.3)
Specialists of Western medicine		192 (41.5)
Practitioner of Chinese medicine - bone-setting		244 (52.7)
Physiotherapist		255 (55.1)
Practitioner of Chinese medicine - acupuncture		221 (47.7)
Practitioner of Chinese medicine (excluding bone-setting / acupuncture)		125 (27)
Others		45 (9.7)

Regarding treatment effectiveness, a significant majority of respondents, 89.8% (n=464), affirmed that the treatment effectively addressed their condition. Additionally, 48.4% (n=249) reported experiencing immediate improvement, while 25.3% (n=130) noted improvements within a few days. About 10.5% (n=54) reported improvement within approximately a week, 6% (n=31) within two weeks, 4.5% (n=23) within one month, and 5.3% (n=27) reported seeing improvements after more than a month of treatment. Table 7 shows the time taken for noticeable improvement in respondents’ condition after beginning chiropractic care.

**Table 7:** The time taken for noticeable improvement in respondents’ condition after beginning chiropractic care n= number of respondents

		n=522 (%)
Immediately		249 (48.4)
Within a few days		130 (25.3)
About a week		54 (10.5)
Within 2 weeks		31 (6)
Within 1 month		23 (4.5)
Over 1 months		27 (5.3)

Nearly all respondents (91.9%, n=480) expressed high levels of satisfaction with their chiropractic treatment experience, and a noteworthy 89.6% (n=468) of the respondents expressed their willingness to recommend chiropractic care to their significant others.

Sick leave authority

Among the respondents, a significant majority (53.6%, n=270) had taken sick leave due to the condition for which they sought chiropractic care. Of those, the majority (64.6%, n=188) had taken one to three days of leave, while 24.7% (n=72) had taken four to seven days, 5.8% (n=17) had taken eight to 14 days, and 4.8% (n=14) had taken 15 days or more. Notably, 65.2% (n=320) of these respondents obtained sick leave certificates from chiropractors.

The survey also addressed a crucial question concerning the potential ramifications if chiropractors were unable to issue sick leave, necessitating patients to seek alternative healthcare professionals for this purpose. The responses unveiled a spectrum of concerns regarding such a scenario, underscoring its multifaceted impact. An overwhelming majority, 85.1% (n=430), expressed deep concern about the financial implications of obtaining sick leave from an alternate source, emphasizing the potential for increased costs. Furthermore, a substantial 82.6% (n=417) of respondents foresaw significant inconveniences in this process, while 72.9% (n=368) voiced apprehensions about extended waiting times. Notably, 59.8% (n=302) were concerned about the possibility of delayed recovery, underscoring the pivotal role of timely sick leave authorization. Moreover, respondents highlighted several potential challenges, including fragmented care (44.8%, n=226), reduced trust in chiropractors (43.8%, n=221), miscommunication (45.5%, n=230), an overburdened healthcare system (56.6%, n=286), and diminished patient autonomy (51.1%, n=258). Table 8 illustrates whether respondents agree that they would be affected by any circumstances if their chiropractor is unable to issue sick leave and they are required to seek another healthcare professional for sick leave.

**Table 8:** Whether respondents agree that they would be affected by any circumstances if their chiropractor is unable to issue sick leave and they are required to seek another healthcare professional for sick leave n= number of respondents

		n=522 (%)
Increased waiting time		368 (72.9)
Require additional costs		430 (85.1)
Delayed recovery		302 (59.8)
Inconvenient		417 (82.6)
Fragmented care		226 (44.8)
Reduce trust in chiropractors		221 (43.8)
Potential for miscommunication		230 (45.5)
Overburdened healthcare system		286 (56.6)
Reduced patient autonomy		258 (51.1)

## Discussion

This survey was conducted to assess the demographics, reasons for seeking chiropractic care, treatment effectiveness, and sick leave authority of chiropractic patients in Hong Kong. The results provide valuable insights into the role of chiropractic care in the Hong Kong healthcare system.

The findings of this study echo previous research, highlighting the predominance of back pain as the primary driver for seeking chiropractic care, aligning with multiple studies [8-11]. Moreover, a substantial portion of respondents in this investigation reported favorable outcomes, corroborating the effectiveness of chiropractic treatment, with many experiencing rapid relief and improvements within days. Furthermore, various studies have delved into the extensive examination of the impact of chiropractic treatment, with some emphasizing its cost-effectiveness in managing musculoskeletal disorders, notably in cases involving low back conditions [11-15].

In this study, a majority of respondents expressed satisfaction with the chiropractic treatment they received. Chiropractors firmly believe that their interventions can act as a preventive measure against the persistence or recurrence of low back pain [16]. Additionally, they emphasize that the treatment's effectiveness hinges on patients' understanding of both low back pain and the corresponding therapeutic approaches [17,18]. Guided by these chiropractic principles, patients often receive preventive advice and detailed explanations of their treatment, leading to high levels of patient satisfaction [11,19].

Despite the evident effectiveness and the high satisfaction reported by patients, a significant portion of respondents in this study sought treatment from other healthcare professionals before considering chiropractic care. The most prevalent reasons for choosing chiropractic care were referrals from others and a desire to explore whether chiropractic interventions could provide relief for their specific conditions. These trends suggest a potential lack of awareness within the community regarding the potential benefits of chiropractic care. This, in turn, could imply that chiropractors are not fully integrated or utilized within the healthcare system in Hong Kong.

Presently, the Hong Kong Hospital Authority reports an extensive waiting period for new case bookings in orthopedics and traumatology, with wait times ranging from 80 to 99 weeks [20]. This delay in assessment undoubtedly has the potential to exacerbate patient conditions and significantly impact their overall quality of life.

Chiropractors, as primary healthcare providers with expertise in diagnosing and managing neuromusculoskeletal complaints [10], offer a viable solution. Given the demonstrated effectiveness and cost-efficiency of chiropractic treatments, it is imperative for authorities to promote the role of chiropractic care to the public. Furthermore, exploring potential public-private partnerships in managing the long wait times for orthopedic patients could alleviate the burden on the healthcare system and improve patient outcomes.

The Hong Kong government's current stance does not extend recognition to chiropractic sick leave certificates. Consequently, patients who acquire such certificates from chiropractors may still find themselves compelled to consult with a general medical practitioner or specialist for other healthcare requirements. Notably, this study revealed that nearly half of the respondents had indeed taken sick leave due to health issues for which they sought chiropractic care. Moreover, the majority of respondents disclosed that their daily lives were significantly affected by their conditions. They expressed their perspectives on the inability of chiropractors to issue sick leave certificates, highlighting various consequences, including inconvenience, concerns about treatment effectiveness, financial strains, and even mental burdens.

The potential benefits of allowing chiropractors to issue sick leave certificates in Hong Kong are multifaceted. Firstly, it can alleviate the burden on other healthcare professionals, particularly general medical practitioners and specialists, by providing an alternative avenue for sick leave authorization. Secondly, it can enhance the accessibility and convenience of obtaining sick leave, especially beneficial for patients balancing work or other commitments. Thirdly, it can contribute to ensuring patients receive timely and suitable care for their conditions. However, the potential challenges of this authority include the risk of misuse or abuse. Nevertheless, this risk can be effectively managed by establishing clear guidelines and regulations governing chiropractic sick leave certification.

The Chiropractic Doctors Association of Hong Kong (CDAHK), being the largest chiropractic organization [21], has recognized the potential issue of misuse and taken proactive steps by issuing a Sick Leave Guideline for Chiropractors in 2014. This guideline provides chiropractors with clear instructions on when sick leave is medically necessary for common conditions falling within their scope of practice [22]. To ensure responsible use of sick leave authority, chiropractors could be subjected to the same oversight and auditing requirements as other healthcare professions that possess this privilege. This approach would enable chiropractors to certify sick leave for the conditions they are qualified to treat while simultaneously minimizing the risks associated with overprescribing or misapplying with this authority.

This study has several limitations worth noting. First, the use of an online survey method and reliance on self-reported data might introduce bias in the findings. Additionally, the study's reliance on descriptive analysis alone could affect the representativeness and generalizability of the results. Furthermore, this research did not investigate the underlying reasons behind the observed treatment effectiveness and patient satisfaction among chiropractic patients. Therefore, further studies are needed to address these limitations and explore the factors contributing to treatment effectiveness and patient satisfaction within the context of chiropractic care in Hong Kong.

## Conclusions

This study provides insights into the characteristics of chiropractic patients in Hong Kong, their experiences with chiropractic care, and their perspectives on chiropractors' authority over sick leave certificates. 

This study reveals that back pain remains the primary reason for seeking chiropractic care, with rapid relief being a common outcome. High patient satisfaction stems from education and preventive approaches. Despite these benefits, many patients first consult other healthcare professionals, highlighting awareness gaps and integration issues within the healthcare system. Hong Kong's lengthy orthopedic wait times suggest a more significant role for chiropractors, potentially through public-private partnerships and increased public awareness. However, the government's stance on chiropractic sick leave certificates is problematic. Many respondents needed sick leave, and the inability of chiropractors to issue certificates raised concerns about convenience, treatment efficacy, finances, and emotional well-being. Allowing chiropractors to authorize sick leave could address these issues, offering timely care and reducing the burden on other healthcare providers. Proper regulation can prevent misuse.

In summary, this study emphasizes the potential of chiropractic care in Hong Kong's healthcare system. Further research and recognizing chiropractors' role in sick leave authorization can enhance the comprehensive care provided by this profession.

## Appendices

**Table 9:** Questionnaire

Demographic questions 個人資料:	
What is your age?你的年齡？	
	Under 15 years old 歲 以下
	15-19 years old 歲
	20-29 years old 歲
	30-39 years old 歲
	40-49 years old 歲
	50-59 years old 歲
	60+ years old 歲以上
What is your gender? 你的性別？	
	Male 男性
	Female 女性
	Other 其他
What is your occupation? 你的職業是什麼？	
	Student 學生
	Full-time employed 全職工作
	Part-time employed 兼職工作
	Self-employed 自僱
	Unemployed 失業
	Home-makers 料理家務者
	Retired 退休
	Other 其他
What is your highest level of education? 你的最高學歷？	
	Primary and below 小學及以下
	Secondary 中學
	Post-secondary 專上教育
Chiropractor consultation 脊醫診治的情況	
How many treatments have you received in the past 12 months? 您在過去 12 個月內接受了多少次治療？	
	1-2 times 次
	3-4 times 次
	5-6 times 次
	7-10 times 次
	11+ times 次
What are your reasons for choosing chiropractic treatment? (Select all that apply) 您選擇脊醫診治的原因? (可選擇多項答案)	
	Relatives / friends’ referral 親友介紹
	Referred by other healthcare professionals 其他醫療專業人士轉介
	Wanted to see whether chiropractic treatment could help alleviate their illness 希望嘗試脊醫診治，以改善病情
	Got to know chiropractor from different media (e.g. Internet / newspaper) 由各種媒體得知脊醫 (例如：互聯網 / 報紙)
	Chiropractic treatment was covered by insurance plan 保險計劃包括脊醫治療
	Others 其他
What was your primary reason for seeking chiropractic care? 您求診脊醫的主要原因是什麼？	
	Back pain 背痛
	Neck pain 頸痛
	Headache 頭痛
	Upper limb pain 上肢痛
	Lower limb pain 下肢痛
	Shoulders pain 肩膊痛
	Other 其他
What parts of your body have received chiropractic treatment? (Select all that apply) 您身體哪些部位接受過脊醫診治? (可選擇多項答案)	
	Waist 腰
	Back 背脊
	Neck 頸
	Lower limbs 下肢
	Upper limbs 上肢
	Shoulders 肩膊
	Head 頭
What is your time lag between injury / illness and first chiropractic treatment? 您傷病與第一次接受脊醫診治相隔的時間	
	Less than 1 week 少於 1 星期
	1 week to less than 2 weeks 1 星期至少於 2 星期
	2 weeks to less than 4 weeks 2 星期至少於 4 星期
	4 weeks to less than 3 months 4 星期至少於 3 個月
	3 months to less than 6 months 3個月至少於 6 個月
	6 months and above 6 個月及以上
How has your condition affected your ability to perform daily activities or work-related tasks? 您的情況對你的日常活動或工作任務產生了什麼影響？	
	Not at all 沒有影響
	Slightly 輕微影響
	Moderately 中等影響
	Significantly 顯著影響
	Unable to perform daily activities or work-related tasks 無法進行日常活動或工作任務
Whether you had received other kinds of treatment before receiving chiropractic treatment ? (Select all that apply) 在未接受脊醫診治之前您是否曾接受其他類型的診治? (可選擇多項答案)	
	Yes 是
	No 否
If you tried other treatments or therapies, please specify the type(s) of treatment or therapy. 如果您有嘗試過其他治療或療法，請說明治療或療法的類型。	
	General medical practitioner of Western medicine 普通科西醫
	Specialist of Western medicine 專科西醫
	Practitioner of Chinese medicine - bone-setting 中醫 - 跌打
	Physiotherapist 物理治療師
	Practitioner of Chinese medicine - acupuncture 中醫 - 針灸
	Practitioner of Chinese medicine (excluding bone-setting / acupuncture) 中醫(不包括跌打 / 針灸)
	Others 其他
How effective do you believe chiropractic care was in addressing your primary health issue? 您認為脊醫治療在解決您的症狀方面有多有效？	
	Very effective 非常有效
	Moderately effective 有效
	Neutral 中性的
	Not very effective 不是很有效
	No effect at all 完全沒有效果
How quickly did you notice an improvement in your condition after beginning chiropractic care? 在接受脊醫治療後，您多久後開始注意到您的狀況得到改善？	
	Immediately 即時舒緩
	Within a few days 數天內
	About 1 week 約一星期
	Within 2 weeks 兩星期內
	Within 1 month 一個月內
	Over 1 month 一個月以上
How satisfied are you with the care you received from your chiropractor? 您對脊醫所提供的治療和護理感到滿意嗎？	
	Very satisfied 非常滿意
	Moderately satisfied 滿意
	Neutral 中性的
	Somewhat dissatisfied 有些不滿意
	Very dissatisfied 非常不滿
How likely are you to recommend chiropractic care to a friend or family member? 您會向朋友或家人推薦脊醫和其療法嗎？	
	Very likely 會
	Moderately likely 可能會
	Neutral 中性的
	Somewhat unlikely 不太會
	Very unlikely 極不會
Have you ever taken sick leave due to the health issue for which you sought chiropractic care? 您有曾因求診脊醫的健康問題而請過病假？	
	Yes 有
	No 沒有
If yes, how many days of sick leave did you take in total? 如果有，您總共請了多少天病假？	
	1-3 days 天
	4-7 days 天
	8-14 days 天
	15 days or more 天或以上
Whether you had obtained sick leave certificates from chiropractors? 您是否曾獲取脊醫所簽發的病假證明書?	
	Yes 有
	No 沒有
Would you agree that you would be affected by the following circumstances if your chiropractor is unable to issue sick leave and you are required to seek another healthcare professional for sick leave? (Select all that apply) 你同意如果你的脊醫無法簽發病假證明書，並需要另外尋求其他醫療專業人員簽發病假證明書，你將面臨以下情況嗎？(可選擇多項答案)	
	Increased waiting time 等候時間增加
	Require additional costs 支付額外費用
	Delayed recovery 延遲康復
	Inconvenience 不便
	Fragmented care 分散的治療
	Reduced trust in chiropractors 降低對脊醫的信任
	Potential for miscommunication 潛在的溝通問題
	Overburdened healthcare system 加重醫療系統負擔
	Reduced patient autonomy 減低病人自主權
	Others 其他
